# Subcapsular sinus macrophages limit acute gammaherpesvirus dissemination

**DOI:** 10.1099/vir.0.000140

**Published:** 2015-08

**Authors:** Bruno Frederico, Brittany Chao, Clara Lawler, Janet S. May, Philip G. Stevenson

**Affiliations:** ^1^​ Division of Virology, Department of Pathology, University of Cambridge, Cambridge, UK; ^2^​ Sir Albert Sakzewski Virus Research Centre, Clinical Medical Virology Centre, School of Chemistry and Molecular Biosciences, Royal Children's Hospital and University of Queensland, Brisbane, Australia

## Abstract

Lymphocyte proliferation, mobility and longevity make them prime targets for virus infection. Myeloid cells that process and present environmental antigens to lymphocytes are consequently an important line of defence. Subcapsular sinus macrophages (SSMs) filter the afferent lymph and communicate with B-cells. How they interact with B-cell-tropic viruses is unknown. We analysed their encounter with murid herpesvirus-4 (MuHV-4), an experimentally accessible gammaherpesvirus related to Kaposi's sarcoma-associated herpesvirus. MuHV-4 disseminated via lymph nodes, and intranasally or subcutaneously inoculated virions readily infected SSMs. However, this infection was poorly productive. SSM depletion with clodronate-loaded liposomes or with diphtheria toxin in CD169–diphtheria toxin receptor transgenic mice increased B-cell infection and hastened virus spread to the spleen. Dendritic cells provided the main route to B-cells, and SSMs slowed host colonization, apparently by absorbing virions non-productively from the afferent lymph.

## Introduction

Persistent virus infections pose major, unsolved health problems. The human gammaherpesviruses Epstein–Barr virus (EBV) and Kaposi's sarcoma-associated herpesvirus (KSHV) colonize B-cells and cause cancers. How they first reach B-cells is hard to define in humans, because infection does not present clinically until it is widespread. Moreover, the narrow species tropisms of these viruses offer little scope for experimental *in vivo* analysis. Such analysis is important nonetheless: vaccination to prevent B-cell binding by cell-free EBV failed to reduce infection rates (Sokal *et al.*, 2007), suggesting that *in vivo* B-cell infection follows routes other than those predominating *in vitro*. Therefore, we must learn from animal models.

Gammaherpesviruses probably colonize all mammals (Saliki *et al.*, 2006; Ehlers *et al.*, 2008; Wilcox *et al.*, 2011). The similar diversities of viruses and their hosts suggest that this colonization preceded most mammalian speciation (McGeoch *et al.*, 2005). Thus, other mammalian gammaherpesviruses can tell us much about human infections. Murid herpesvirus-4 (MuHV-4) is a widely studied, KSHV-like gammaherpesvirus of mice that realistically infects inbred, laboratory mouse strains, and so provides an opportunity to define *in vivo* key features of host colonization (Stevenson *et al.*, 2009; Barton *et al.*, 2011). Mucosally delivered MuHV-4 first reaches B-cells in lymph nodes (LNs) via a CD11c^+^ myeloid intermediary (Gaspar *et al.*, 2011). Viruses, being immotile, must spread via host pathways, and CD11c^+^ cell dependence suggests a role for dendritic cells (DCs) in MuHV-4 transfer to B-cells, analogous to the classical DC functions of antigen transport and presentation (Cella *et al.*, 1997). However, subcapsular sinus macrophages (SSMs) also express CD11c (Gray and Cyster, 2012). These cells are sessile and capture antigens from the afferent lymph rather than from peripheral tissues. Small soluble antigens exit the subcapsular sinus via conduits (*et al.*, 2009), and DCs migrate into LNs between SSMs (Braun *et al.*, 2011); SSMs capture intermediate-sized antigens, including immune complexes, infected-cell debris and cell-free virions. They can transfer these antigens to B-cells (Carrasco and Batista, 2007; Junt *et al.*, 2007; Phan *et al.*, 2007), and possibly also to LN DCs (Hickman *et al.*, 2008). Analogous splenic marginal zone macrophages (MZMs) capture blood-borne antigens (Mebius *et al.*, 2004) and transfer MuHV-4 to marginal zone B-cells (Frederico *et al.*, 2014). Therefore, gammaherpesviruses could use either migratory DCs or SSMs to reach B-cells.

In addition to antigen presentation, SSMs have direct defensive functions. For example, they capture injected vesicular stomatitis virus from the afferent lymph and restrict its access to neurons (Iannacone *et al.*, 2010). MZMs also have a defensive role, limiting the blood-borne spread of lymphocytic choriomeningitis virus (Seiler *et al.*, 1997), murine cytomegalovirus (Hamano *et al.*, 1998), vesicular stomatitis virus (Oehen *et al.*, 2002) and West Nile virus (Winkelmann *et al.*, 2014). Like DCs (Moretta, 2002), SSMs interact with NK cells (Garcia *et al.*, 2012) and are a prominent site of interferon-α/β induction (Sandberg *et al.*, 1994). Lymph-borne viruses must evade such defences to engage in productive replication.

Virus evasion is often host restricted, and so may be compromised in xenogenic infections. The natural host of MuHV-4 appears to be yellow-necked mice (*Apodemus flavicollis*) (Kozuch *et al.*, 1993) rather than the house mice (*Mus domesticus*) from which inbred laboratory strains are derived. However, MuHV-4 immune evasion appears to function in laboratory mice: CD8^+^T-cell evasion functions defined for KSHV in human cells are retained by the homologous MuHV-4 genes in murine cells (Stevenson *et al.*, 2000), as are chemokine binding (Parry *et al.*, 2000), IFN evasion (Hwang *et al.*, 2009), complement evasion (Kapadia *et al.*, 1999), virus episome maintenance (Habison *et al.*, 2012), apoptosis and autophagy inhibition (Liang *et al.*, 2015), modulation of B-cell receptor signalling (Pires de Miranda *et al.*, 2013), disassembly of nuclear bodies (Gaspar *et al.*, 2008) and superantigen-like T-cell stimulation (Evans *et al.*, 2008). Indeed, there is no clear example of a predicted MuHV-4 evasion protein being non-functional in laboratory mice. In addition, MuHV-4 persists in laboratory mice without causing disease unless there is immune suppression, and is transmitted through sexual contact (François *et al.*, 2013). It is clearly attenuated in bank voles (François *et al.*, 2010), and a preliminary study of field mice (Hughes *et al.*, 2010), a close relative of yellow-necked mice, showed no convincing difference in host colonization from laboratory mice. Therefore, MuHV-4 provides a suitable tool to define in laboratory mice how gammaherpesviruses colonize lymphoid tissue.

## Results

### A MuHV-4 footpad infection model

**Fig. 1.**
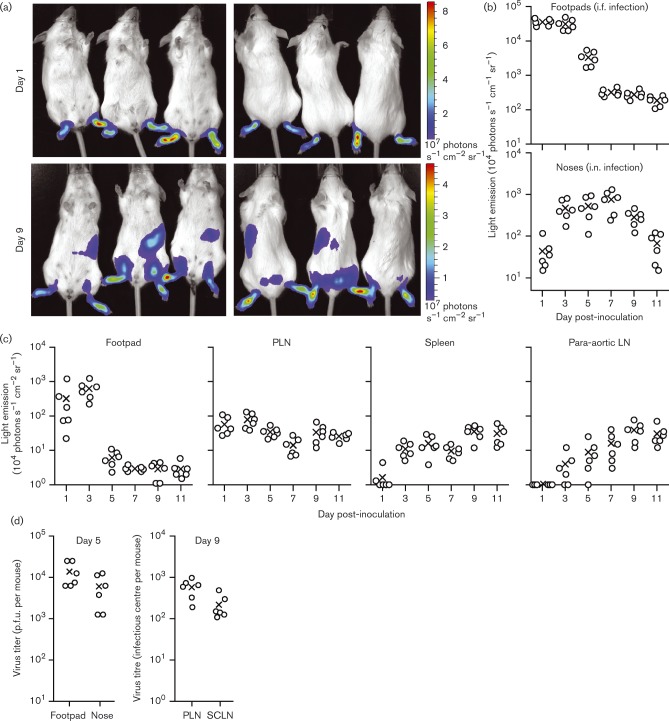
MuHV-4 infection via footpad inoculation. (a) BALB/c mice were infected i.f. with luciferase-expressing MuHV-4 (MHV-LUC; 10^5^ p.f.u.). Infection was monitored by intraperitoneal (i.p.) luciferin injection and charge-coupled device camera scanning of light emission. Representative images are shown at days 1 and 9 of infection. Abdominal signals at day 9 came from the spleen. (b) Quantification of live imaging for mice given MHV-LUC (10^5^ p.f.u.) either i.f. (footpad signals) or i.n. (nose signals), showing the comparative signal strengths and kinetics of infection. ○, Individual mice; × , means. Footpad signals were significantly higher than nose signals at days 1–5 (*P* < 0.01), but not at days 7–11 (*P* >0.05). (c) To identify the sources of live imaging signals, mice were infected i.f. with MHV-LUC as in (a) and then dissected at each time point to image the relevant organs *ex vivo*. Footpads and PLNs were positive from day 1, whereas most spleens and para-aortic LNs were positive only from day 3. The *y*-axis baseline corresponds to the lower limit of assay sensitivity. (d) BALB/c mice were infected i.n. or i.f. with MHV-LUC as in (b). After 5 days, footpads, (i.f. infection) and noses (i.n. infection) were compared for infectious virus titre by plaque assay. After 9 days, PLNs (i.f. infection) and SCLNs (i.n. infection) were compared for total recoverable virus titre by infectious centre assay. Titres were higher after i.f. infection but not significantly so (*P* >0.05).

Most MuHV-4 studies have delivered virions intranasally (i.n.), from where they must cross an epithelial surface to reach the lymphatics. Most SSM studies have delivered antigens more directly to lymphatics by subcutaneous inoculation. To reconcile our infection studies with those of other antigens, we established a new model of intra-footpad (i.f.) MuHV-4 inoculation (Fig. 1). Live imaging of virus-expressed luciferase (MHV-LUC) (Fig. 1a, b) showed strong footpad signals from 1 day after i.f. inoculation. By contrast, nasal infection peaked at 5–7 days after i.n. inoculation. Peak live imaging signals were 30-fold stronger from footpads than from noses (Fig. 1b).

MHV-LUC reaches the superficial cervial LN (SCLN) 4–6 days after i.n. inoculation (Gaspar *et al.*, 2011). It was not possible to distinguish footpad from popliteal LN (PLN) infection by live imaging, but imaging of dissected organs (Fig. 1c) showed rapid PLN colonization after i.f. inoculation, with strong signals at day 1, followed by spread to the para-aortic LN and spleen. At 5 days post-inoculation, comparable titres of infectious virus were recovered from footpads (i.f.) and noses (i.n.); and at 9 days post-inoculation, comparable titres of reactivatable virus were recovered from PLNs (i.f.) and SCLNs (i.n.) (Fig. 1d). Therefore, footpad infection was highly productive and spread rapidly to LNs.

### *In situ* identification of early PLN infection

**Fig. 2.**
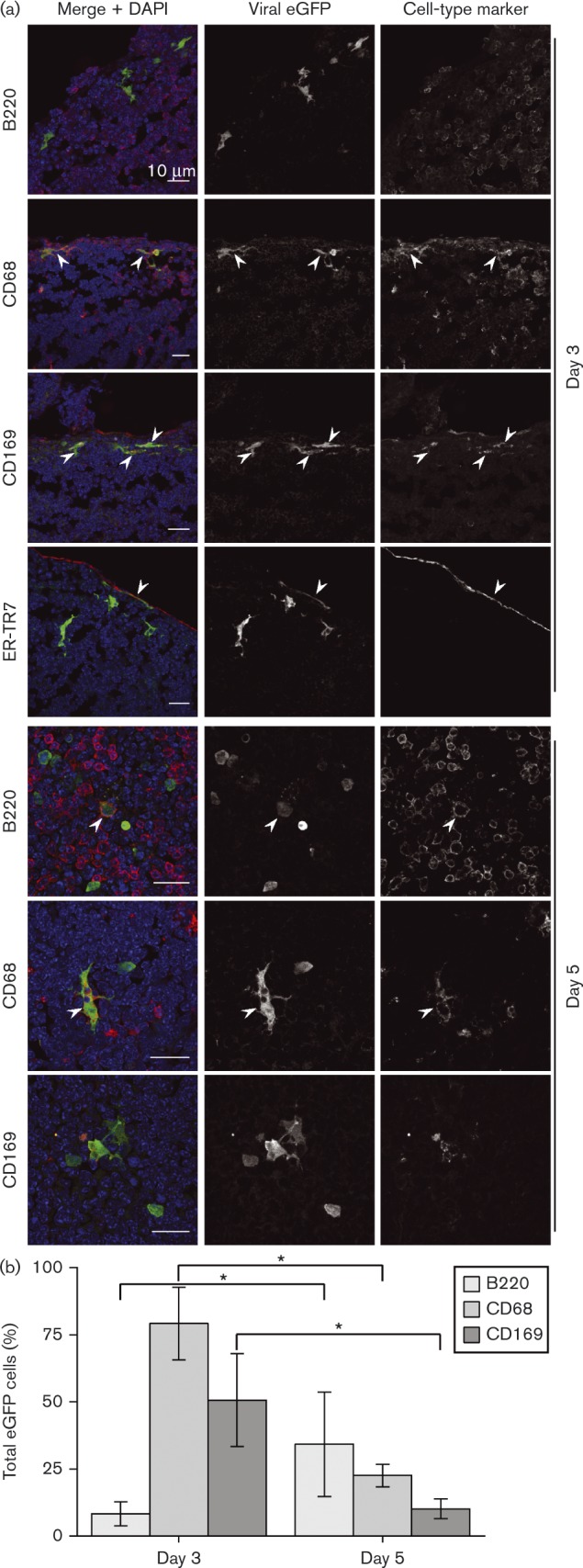
Early colonization of PLNs by i.f.-inoculated MuHV-4. (a) C57BL/6 mice were infected i.f. with MHV-GFP. Sections of PLNs harvested 3 and 5 days later were stained for virus-expressed eGFP (green in merge) and for cell-type markers (red in merge). Nuclei were stained with DAPI (blue). The images are representative of at least six mice per time point. Arrowheads show examples of green/red co-localization. (b) eGFP^+^ cells were counted for five mice (three sections per mouse). Bars show means ± sem for eGFP^+^ cells expressing each marker (co-localization with ER-TR7 was < 2  %). From day 3 to day 5, eGFP^+^B220^+^ cell numbers increased significantly and eGFP^+^CD68^+^ and CD169^+^eGFP^+^ cell numbers decreased significantly (*P < 0.02).

To determine how MuHV-4 spreads through the PLN, we inoculated C57BL/6 mice i.f. with MHV-GFP, which expresses eGFP from an EF1α promoter independently of lytic gene expression (May & Stevenson, 2010) and so reveals both lytically and latently infected cells (Fig. 2). We identified infected cells by immunostaining tissue sections. Although flow cytometry provides potentially more precise quantification, it has significant limitations for analysing early MuHV-4 infection. Firstly, with too few cells involved to form clear populations, flow cytometry struggles to distinguish positive staining from autofluorescence. Secondly, key myeloid populations are recovered poorly from LN homogenates. Thus, flow cytometry shows B-cell infection by EF1α-eGFP MuHV-4 but does not show convincingly the preceding myeloid infection, despite this being clear on tissue sections (Gaspar *et al.*, 2011). Thirdly, the anatomical site of myeloid cells may be more important than their expression of correlative markers: for example, CD169 is not unique to SSMs and is largely redundant for lymph filtration (Oetke *et al.*, 2006). Homogenization of tissues therefore loses important information. It can lead also to non-physiological cell interactions and marker acquisition (Gray & Cyster, 2012).

At day 3 post-inoculation, PLN sections rarely showed eGFP^+^B-cells (B220^+^); most eGFP^+^ cells were CD68^+^ myeloid cells, and most of these were CD169^+^ SSMs. Some associated closely with the reticular supporting network were stained by mAb ER-TR7, but ER-TR7^+^ fibroblasts were rarely eGFP^+^. By day 5, infected B-cells were more evident – presumably reflecting virus-driven lymphoproliferation – and myeloid infection had declined. CD169 staining was also reduced generally. Many eGFP^+^ cells had a lymphocytic morphology and were in B-cell-rich areas, but showed weak or absent B220 staining by immunofluorescence. This is consistent with the idea that many acutely infected B-cells differentiate into plasma cells (Collins *et al.*, 2009), as these downregulate B220 (Jensen *et al.*, 1989).

**Fig. 3.**
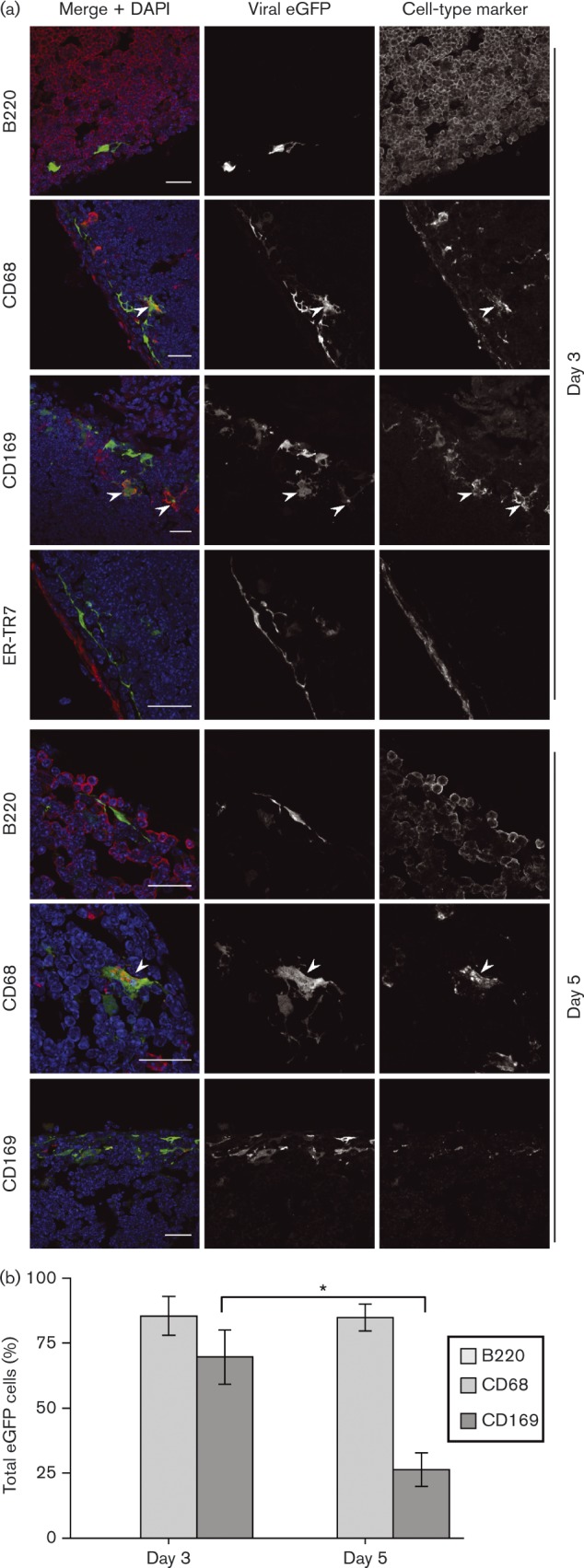
PLN infection by i.f. replication-deficient MuHV-4. (a) C57BL/6 mice were infected i.f. with replication-deficient MHV-GFP (ORF50^− ^). Sections of PLN harvested 3 and 5 days later were stained for viral eGFP (green in merge) and cell-type markers (red in merge). Nuclei were stained with DAPI (blue). The images are representative of six mice per time point. Arrows show examples of green/red co-localization. Scale bar = 10 μm. (b) EGFP^+^ cells were counted for five mice (three sections per mouse). Bars show mean ± sem for eGFP^+^ cells expressing each marker (co-localization with ER-TR7 was < 2 %). From day 3 to day 5, CD169^+^eGFP^+^ cell numbers decreased significantly (*P < 0.02). This was associated with a general loss of CD169 staining, seen also with wild-type infection.

To identify the PLN cells infected directly by i.f. MuHV-4, we used an MHV-GFP derivative that lacked the essential lytic transactivator encoded by ORF50 (Milho *et al.*, 2009). Infection was visualized again by staining for virus-expressed eGFP (Fig. 3). No eGFP^+^ cells were B220^+^ at day 3; most were CD169^+^CD68^+^ and close to the LN capsule defined by ER-TR7 staining. Whilst ORF50^+^ infection progressed to B-cells by day 5 (Fig. 2), ORF50^− ^ infection did not. Again, CD169 staining was reduced relative to day 3, but on the basis of cell morphology and CD68 staining, essentially all eGFP^+^ cells were myeloid. Therefore, i.f. MuHV-4 directly infected SSMs and not B-cells.

### Comparison with SCLN infection

**Fig. 4.**
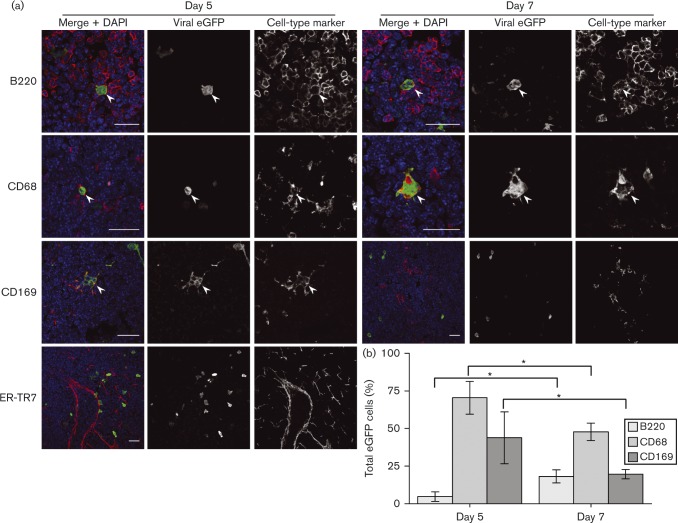
Early SCLN infection by i.n. MuHV-4 inoculation. (a) C57BL/6 mice were infected i.n. with MHV-GFP (10^5^ p.f.u.). Sections of SCLNs harvested 5 and 7 days later were stained for viral eGFP (green in merge) and cell-type markers (red in merge). Nuclei were stained with DAPI (blue). The images are representative of six mice per time point. Arrows show examples of green/red co-localization. Scale bar = 10 μm. (b) eGFP^+^ cells were counted for five mice (three sections per mouse). Bars show mean ± sem for eGFP^+^ cells expressing each marker (co-localization with ER-TR7 was < 2 %). From day 5 to day 7, eGFP^+^B220^+^ cell numbers increased significantly and eGFP^+^CD68^+^ and CD169^+^eGFP^+^ cell numbers decreased significantly (*P < 0.02).

Few eGFP^+^ cells were evident in SCLNs 3 days after i.n. (ORF50^+^) MHV-GFP inoculation, so we analysed them at days 5 and 7 (Fig. 4). Even at day 5, SCLNs contained fewer eGFP^+^ cells than day 3 PLNs (approx. one-third of the total number), and no eGFP^+^ cells were seen in SCLNs of mice inhaling replication-deficient MHV-GFP (data not shown), arguing that SCLN colonization was delayed relative to PLNs because i.n. MuHV-4 must replicate to reach the lymphatics. As with day 3 i.f. infections, most cells infected 5 days after i.n. MuHV-4 inoculation were CD68^+^. Approximately 50 % of these were CD169^+^. Therefore, SSMs were a prominent infection target after both i.f. and i.n. inoculations. From day 5 to day 7, the proportion of eGFP^+^ cells that were B220^+^ increased significantly and the proportion that were CD68^+^ or CD169^+^ decreased. Thus, whilst i.n. and i.f. MuHV-4 showed marked kinetic differences in LN colonization, both progressed from myeloid to B-cell infection.

### Infection tracked by floxed virus tagging

**Fig. 5.**
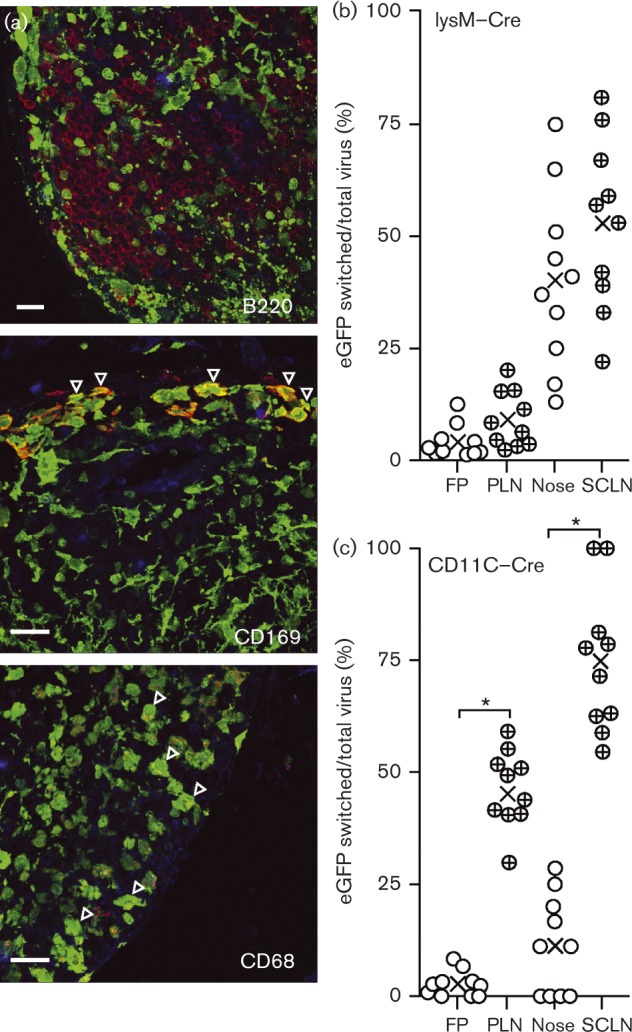
MuHV-4 genetic switching by Cre in LNs of Cre transgenic mice. (a) LN sections from naive LysM–Cre × Ai6–ZsGreen mice were stained for cell markers (red). Cre-triggered ZsGreen1 expression was visualized directly (green). Nuclei were stained with DAPI (blue). Arrows show examples of red/green co-localization, including CD169^+^ cells around the subcapsular sinus. Bars, 10 μm. (b) LysM–Cre mice were infected i.f. or i.n. with MHV-RG (10^5^ p.f.u.). Viruses recovered from footpads (FP) and PLNs 5 days after i.f. infection, and from noses and SCLNs 7 days after i.n. infection were typed for Cre-mediated switching from red to green fluorescence. The percentage switched was calculated as 100 × green p.f.u./(green pf.u. + red p.f.u.). ○ and ⊕, Individual mice; × , means. PLN switching was not significantly different from that in the footpad, and SCLN switching was not significantly different from that in noses (*P* >0.05). (c) CD11c-Cre mice were infected i.f. or i.n. with MHV-RG, and recovered viruses were typed for Cre-mediated switching as in (b). Switching was significantly greater in PLNs than in the footpad, and significantly greater in SCLNs than in noses (*P < 0.001).

The productivity of SSM infection was hard to gauge by immunostaining: few cells expressed lytic antigens, but this is only a snapshot of infection, and the quantitative relationship between lytic antigen expression and virus production is unclear. Therefore, we gauged productivity instead by Cre-mediated virus marking, infecting lysM–Cre mice with MuHV-4 in which Cre switches the fluorochrome expression cassette from mCherry (red) to eGFP (green) (MHV-RG) (Frederico *et al.*, 2012). LysM is expressed predominantly in macrophages and granulocytes (Clausen *et al.*, 1999). In lysM–Cre mice crossed with the Ai6–ZsGreen1 reporter strain, in which Cre activates ZsGreen1 expression from a tissue-non-selective promoter (Madisen *et al.*, 2010), CD169^+^ and CD68^+^ LN myeloid cells but not B220^+^B-cells were ZsGreen^+^ (Fig. 5a). Therefore, SSMs expressed lysM, and we could use MHV-RG switching to estimate SSM infection productivity in lysM–Cre mice.

We infected lysM–Cre mice with MHV-RG either i.n. or i.f., recovered viruses 5 days later from noses and SCLNs (after i.n. inoculation) or from footpads and PLNs (after i.f. inoculation) and then typed these viruses for eGFP and mCherry expression (Fig. 5b). I.n. MHV-RG showed substantial switching (∼50 %) from mCherry^+^ (input) to eGFP^+^ (recombined by Cre). However, this occurred in noses rather than in SCLNs, as viruses recovered from SCLNs showed no significant increase in switching above those recovered from noses. I.f. MHV-RG showed little switching in either footpads or PLNs (mean < 10 %). By contrast, MHV-RG given i.n. to CD11c-Cre mice showed substantial switching (75 %) between noses and SCLNs, and moderate switching (45 %) between footpads and PLNs (Fig. 5c). SSMs express CD11c at only low levels (Gray & Cyster, 2012). Therefore, they seemed unlikely to account for MHV-RG switching, being greater in CD11c-Cre than in lysM-Cre mice. Rather, productive virus entry into LNs appeared to involve cells with a DC-like (CD11c^hi^lysM^lo^) rather than macrophage-like (CD11c^lo^lysM^hi^) profile.

### Effect of SSM depletion on host colonization

**Fig. 6.**
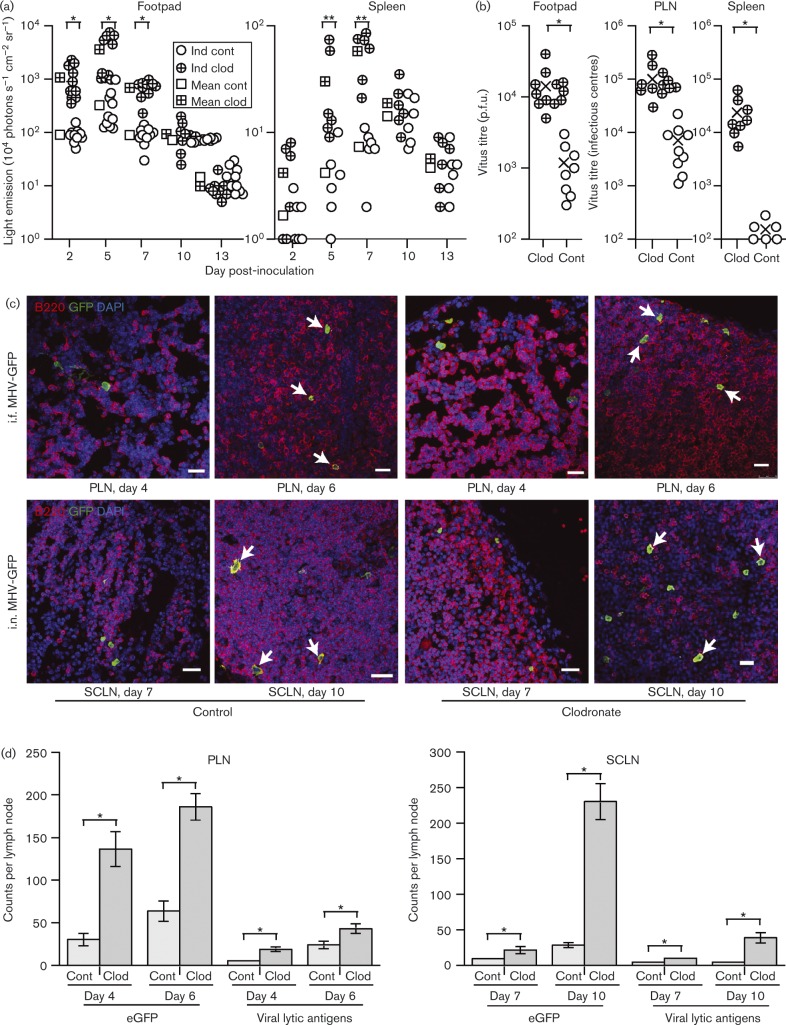
Effect of lipsomal clodronate on the spread of i.f. and i.n. MuHV-4. (a) BALB/c mice were depleted of phagocytic cells by i.f. liposomal clodronate (clod), 3 and 5 days before i.f. MHV-LUC (10^5^ p.f.u.) inoculation. Control mice (cont) were given virus without depletion. Infection was then tracked by intraperitoneal (i.p.) luciferin injection and live imaging of light emission. Individual mice (ind) and mean values are shown. The *y*-axis baseline corresponds to the lower limit of assay detection. Clodronate treatment significantly increased light emission from footpads at days 2–7 post-inoculation (*P < 0.01), and from spleens at days 5–7 (**P < 0.02) post-inoculation. (b) Mice were infected as in (a), and titrated 7 days later for infectious virus by plaque assay (footpads) and for total virus by infectious centre assay (PLNs and spleens). ○ and ⊕, Individual mice; × , means. Clodronate increased infection in all sites (*P < 0.01). (c) C57BL/6 mice were depleted or not of SSMs by subcutaneous injection of clodronate-loaded liposomes and then given MHV-GFP either i.n. or i.f. For i.n. infections, liposomes were injected around the snout; for i.f. infections, liposomes were injected into footpads. LN sections were examined for B-cell infection by co-staining for B220 (red) and virus-expressed eGFP (green). Nuclei were stained with DAPI (blue). PLNs were examined 4 and 6 days after i.f. inoculation; SCLNs were examined 7 and 10 days after i.n. inoculation. Arrows show examples of eGFP^+^B220^+^B-cells. Bars, 10 μm. (d) Quantification of eGFP^+^ cells showed similar infection kinetics between clodronate-treated and control mice, but significantly higher totals in the treated mice (*P < 0.01). Each bar shows mean ± sem of counts from six mice, examining six sections per mouse. Lytic antigen staining also showed a significant increase with clodronate (*P < 0.01). Lytic antigen^+^ cell numbers were approximately 20 % of eGFP^+^ cell numbers regardless of clodronate. The proportion of eGFP^+^ cells staining for B220 was also not significantly different between treated and control mice. Lytic antigen^+^B220^+^ cells were too few for accurate counts.

Clodronate-loaded liposomes deplete SSMs (van Rooijen & Sanders, 1994). Therefore, to determine how SSM infection affects host colonization by MuHV-4, we gave BALB/c mice i.f. clodronate-loaded or control liposomes, 3 and 5 days before i.f. MHV-LUC. Live imaging (Fig. 6a) showed early increases in both footpad and spleen luciferase signals after clodronate treatment. We also measured virus titres at 7 days post-inoculation (Fig. 6b). Footpad, PLN and spleen titres were all significantly higher in clodronate-treated mice, with spleens showing the greatest difference.

Next, we depleted C57BL/6 mice of PLN SSMs using i.f. liposomal clodronate and infected them i.f. with MHV-GFP (Fig. 6c). PLN sections showed similar distributions of eGFP^+^ infected cells in depleted and control mice. Notably, clodronate did not compromise virus entry into the PLN areas marked by B220 staining. The same was true of SCLNs, when SSMs were depleted by injecting clodronate-loaded liposomes subcutaneously around the snout, 3 and 5 days before i.n. MHV-GFP inoculation (Fig. 6c). Enumeration of eGFP^+^ cells across multiple sections showed significantly more LN infection after clodronate treatment, for both i.f. and i.n. virus inoculations (Fig. 6d). Viral lytic antigen staining was also increased, although relatively few cells expressed lytic antigens compared with eGFP^+^. Clodronate treatment did not significantly affect the ratio of eGFP^+^:lytic antigen^+^ cells in either PLNs or SCLNs.

**Fig. 7.**
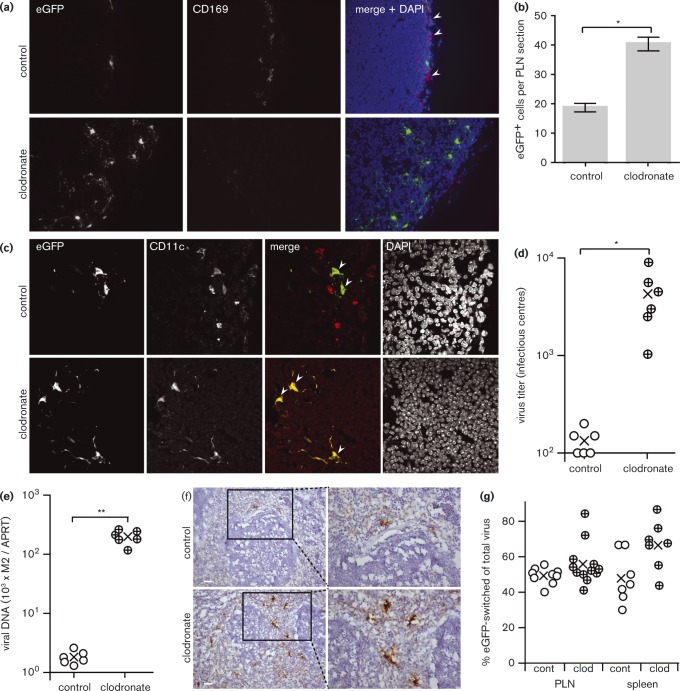
MuHV-4 spread to the spleen in mice depleted of SSMs. (a) C57BL/6 mice were depleted or not of SSMs by i.f. clodronate-loaded liposomes, 3 and 5 days before i.f. inoculation of replication-deficient (ORF50^− ^) MHV-GFP (10^5^ p.f.u.). Four days later, PLN sections were stained for viral eGFP (green in merge) and CD169 (red). Nuclei were stained with DAPI (blue). Arrowheads show example CD169^+^ cells, which were essentially absent after clodronate treatment. (b) Mean ± sem counts of eGFP^+^ cells from three sections each of three mice per group showed a significant increase with clodronate (*P < 0.01). (c) C57BL/6 mice were given clodronate-loaded liposomes i.f. or not and then infected with ORF50^− ^ MHV-GFP i.f. as in (a). Four days later, PLN sections were stained for viral eGFP (green in merge) and CD11c (red). Nuclei were stained with DAPI (blue). Arrowheads show examples of red/green co-localization. (d) C57BL/6 mice were given clodronate-loaded liposomes i.f. or not as in (a) and then infected i.f. with WT MuHV-4 (10^5^ p.f.u.). Virus titres in spleens were determined 3 days later by infectious centre assay. ○ and ⊕, Individual mice; × , means. Clodronate treatment significantly increased viral titres (*P < 0.001). (e) Mice were given liposomal clodronate or not and then infected i.f. as in (d). Viral DNA loads were quantified by PCR (M2 gene) and normalized by PCR of cellular DNA (APRT gene). ○ and ⊕, Individual mice; × , means. Clodronate treatment significantly increased viral DNA loads (**P < 0.001). (f) Mice were given liposomal clodronate or not and then infected i.f. as in (d). Spleen sections were stained for MuHV-4 lytic antigens (brown) and counterstained with haematoxylin (blue). Bars, 50 μm. The boxed regions in the left-hand panels are shown at higher magnification in the right-hand panels. Clodronate treatment significantly increased the number of viral antigen^+^ cells (*P* < 0.01) (see main text for numbers). (g) CD11c-Cre mice were depleted or not of SSMs as in (a) and then infected i.f. with the Cre-sensitive switching virus MHV-RG. Seven days later, virus was recovered from PLNs and spleens by infectious centre assay and typed as mCherry^+^ (native) or eGFP^+^ (switched). ○ and ⊕, Individual mice; × , means. Clodronate treatment increased total virus titres 10–100-fold. It also increased the proportion of switched virus relative to controls but not by a significant amount (*P*>0.05).

Clodronate treatment also increased significantly PLN infection by i.f.-inoculated ORF50^− ^ MuHV-4 (Fig. 7a, b). Therefore, PLN infection increased independently of the increase in footpad infection seen with WT MuHV-4 in Fig. 6(a, b). In contrast to the depletion of CD169^+^ SSMs by clodronate (Fig. 7a), CD11c^hi^ dendritic cells were maintained and were abundantly infected (Fig. 7c). WT MuHV-4 dissemination to the spleen, as measured by virus titres (Fig. 7d) and DNA loads (Fig. 7e) 3 days after i.f. inoculation, increased significantly when SSMs were depleted. Staining spleens for lytic antigens (Fig. 7f) showed a significant increase (*P* < 0.01) in virus colonization of the marginal zone: control mice had 1.8 ± 1.1 and clodronate-treated mice had 17.1 ± 9.6 lytic antigen^+^ cells per follicle (mean ± sem, six sections from three mice). Therefore, LN SSMs limited acute MuHV-4 dissemination to the spleen.

We also tested how SSM depletion affected MHV-RG switching in CD11c-Cre mice by giving clodronate-loaded liposomes i.f. or not, 3 and 5 days before i.f. MHV-RG (Fig. 7g). Viruses recovered from PLNs and spleens 7 days later were typed for eGFP (switched) and mCherry (unswitched) fluorochrome expression. Clodronate treatment did not reduce switching – indeed, it increased switching, although the increase was not statistically significant. As DCs resist depletion by liposomal clodronate (van Rooijen & Sanders, 1994), this result was consistent with DCs being more important than SSMs for MuHV-4 transfer to B-cells.

### Effect of CD169^+^ cell depletion on host colonization

**Fig. 8.**
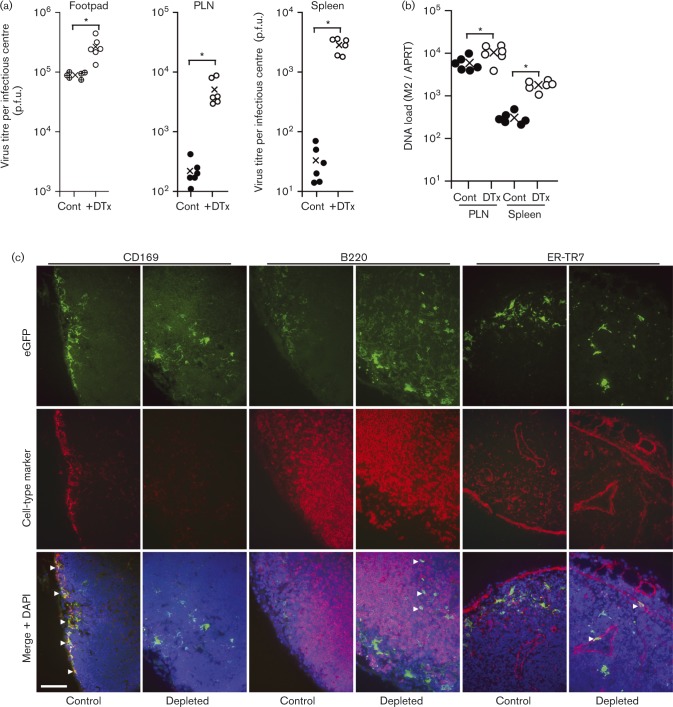
Effect of CD169^+^ cell depletion on the spread of i.f. MuHV-4. (a) CD169-DTR mice were depleted or not of CD169^+^ cells by i.p. DTx injection and then infected i.f. with MHV-GFP. Three days later, virus was titrated in footpads by plaque assay and in PLNs and spleens by infectious centre assay. ○, ⊕ and •, Individual mice; × , means. DTx treatment significantly increased virus titres in each site (*P < 0.01), with the increase in splenic titres being significantly greater than in footpads or PLN (*P* < 0.01). (b) Mice were depleted or not and then infected as in (a). Three days later, viral DNA loads were assayed in PLNs and spleens by PCR (M2 gene) and normalized by the cellular DNA load of each sample, assayed in parallel (APRT gene). DTx treatment significantly increased viral DNA loads in PLNs (**P < 0.05) and spleens (*P < 0.01). (c) Mice were depleted or not and then infected as in (a). Three days later, PLN sections were stained for virus-expressed eGFP (green) and for cell-type-specific markers (red). Nuclei were stained with DAPI (blue). Arrows show examples of green/red co-localization. Bar, 50 μm.

Phagocyte depletion by liposomal clodronate causes inflammation. Thus, virus replication could be affected by either the depletion itself or its secondary effects. As an alternative approach, therefore, we depleted CD169^+^ cells by treating CD169–diphtheria toxin receptor mice (CD169^+/DTR^) with diphtheria toxin (DTx). This also causes inflammation (Lawler *et al.*, 2015). However, it is more cell-type specific and more rapidly effective than clodronate treatment, so the inflammation is much less. We injected DTx intraperitoneally (i.p.), 1 and 2 days before i.f. challenge with MHV-GFP (Fig. 8). Control CD169^+/DTR^ mice were infected without depletion. After 2 days, we titrated the virus in footpads by plaque assay and that in PLNs and spleens by infectious centre assay (Fig. 8a). DTx treatment increased the virus titres in all sites, with the greatest increase in spleens. Quantification of viral DNA in PLNs and spleens (Fig. 8b) confirmed that infection increased. Clodronate treatment depletes phagocytic cells locally, whereas DTx depletes all CD169^+^ cells, so MZMs and SSMs may both have contributed to the greater splenic infection of DTx-treated mice. Nonetheless, it was clear that CD169^+^ cell depletion increased MuHV-4 systemic spread.

We also analysed infection by immunostaining depleted and control PLNs for virus eGFP expression (Fig. 8c). Total eGFP^+^ cell numbers were similar between depleted and control mice. However, their distribution changed: in control mice, eGFP^+^ cells were outside B220^+^ follicles and were largely CD169^+^ (Fig. 8c, CD169, control, arrows), whereas depleted mice, which lost CD169 staining (Fig. 8c, CD169), also had eGFP^+^ cells in B-cell follicles (Fig. 8c, B220, depleted, arrows) and in ER-TR7^+^ fibroblasts (Fig. 8c, ER-TR7, depleted, arrows). As MHV-GFP expresses eGFP independently of virus replication, the higher PLN virus titres of DTx-treated mice (Fig. 8a) despite similar eGFP^+^ cell numbers suggested that their infection was more productive, i.e. that the CD169^+^ cells infected in control mice produced virus poorly. Thus, MHV-RG switching, clodronate treatment and CD169^+^ cell depletion all argued that SSM infection restricts rather than promotes MuHV-4 spread to B-cells.

## Discussion

Peripheral antigens follow two main routes to B-cells: capture and transport by DCs, and bulk lymphatic flow to SSMs. Viruses follow host-defined paths, so gammaherpesviruses could reach B-cells potentially via either DCs or SSMs. These routes could also overlap, for example by SSM–DC antigen transfer (Hickman *et al.*, 2008). We examined the interaction between MuHV-4 and SSMs. MuHV-4 can use a range of myeloid intermediaries to infect B-cells *in vitro* (Frederico *et al.*, 2012), but SSMs did not provide a major route to B-cells *in vivo* after either footpad or upper respiratory tract inoculation. SSMs were readily infected, but this infection appeared to be poorly productive and SSM depletion increased infection spread. These data supported the idea that MuHV-4 reaches B-cells primarily via DCs.

Virus delivery by subcutaneous injection bypasses the need for replication to penetrate epithelial barriers. The limited subcutaneous space of mouse footpads means that most of a 50 μl i.f. injection must pass rapidly along lymphatics to SSMs. The inflammatory response to mucosal infection also promotes lymphatic flow but develops only after virus replication and spread. Thus, for virions at an intact mucosal surface, early DC migration may offer a faster route to B-cells than bulk lymphatic flow. The greater switching of i.n. than i.f. MHV-RG in CD11c-Cre LNs argued that peripheral replication promotes DC infection. This may also be important for early immune priming by mucosal MuHV-4 (Mount *et al.*, 2010). SSM infection should reinforce DC-driven responses, but a more important SSM function may be to contain locally the large amounts of virus produced by peripheral replication. Subcutaneous injection models lymphatic antigen delivery after peripheral replication, but its rapidity and directness – as seen by i.f. replication-deficient MuHV-4 infecting SSMs – could increase the role of SSMs in immune priming. Such effects must be considered when extrapolating experimental data to natural infections.

CD169^+^ LN SSMs are analogous to CD169^+^ metallophilic splenic MZMs: both capture antigens – from the lymph and blood, respectively – and transfer them to B-cells. However, whilst SSM infection was poorly productive, CD169^+^ MZMs support MuHV-4 lytic gene expression and pass infection to marginal zone B-cells, with splenic colonization proceeding via lysM^+^ rather than CD11c^+^ cells (Frederico *et al.*, 2014). That splenic infection was maintained in mice depleted of CD169^+^ cells was unsurprising, as MuHV-4 productively infects CD169^− ^MARCO^+^ splenic MZMs (Frederico *et al.*, 2014). Depleting both MZM populations with i.p. liposomal clodronate (van Rooijen & Sanders, 1994) also failed to stop splenic infection because MuHV-4 can reach B-cells via F4/80^+^ red-pulp macrophages (B. Frederico and P. G. Stevenson, unpublished data). Thus, MuHV-4 can exploit a range of lysM^+^ splenic macrophages to reach marginal zone B-cells. The lower productivity of SSM infection could reflect differences in the innate immune response: subcapsular sinuses and the splenic marginal zone are both prominent interferon-α/β transcription sites, but marginal zone responses may be tempered by post-translational regulation (Honke *et al.*, 2011). There may be also important differences in how MuHV-4 reaches SSMs and MZMs. For example, because MZM infection occurs later in host colonization, virions could reach them as immune complexes attached to erythrocyte complement receptors (Cornacoff *et al.*, 1983). Resolving these possibilities requires further investigation. The present data show that i.f. and i.n. MuHV-4 readily infect SSMs but depend more on DC infection for spread. By being accessible to infection and then propagating it poorly, SSMs restricted acute virus spread.

## Methods

### Mice

BALB/c, C57BL/6J (Harlan, UK, or Animal Resources Centre, Western Australia), CD11c-Cre (Caton *et al.*, 2007), LysM-Cre (Clausen *et al.*, 1999), Ai6-ZsGreen1 (Madisen *et al.*, 2010), and CD169-DTR mice (Asano *et al.*, 2011) were maintained in the Department of Pathology, University of Cambridge, UK, or the Herston Medical Research Centre, Queensland, Australia, and used when 6–12 weeks old. Animal experiments were approved by the Cambridge University ethical review board, the UK Home Office (Project Licence 80/2538) and the University of Queensland Animal Ethics Committee in accordance with Australian National Health and Medical Research Council guidelines. Viruses were administered (10^5^ p.f.u.) either subcutaneously into the footpad under isoflurane anaesthesia (i.f., 50 μl) or by allowing spontaneous inhalation without anaesthesia (i.n., 5 μl) (Tan *et al.*, 2014). For luciferase imaging, mice were given 2 mg d-luciferin i.p., anaesthetized by isoflurane inhalation and monitored for light emission by charge-coupled device camera scanning (Xenogen IVIS-200). To deplete phagocytic cells, mice were injected i.f. twice with 50 μl clodronate-loaded liposomes (http://clodronateliposomes.org/) (van Rooijen & Sanders, 1994), 3 and 5 days before infection. To deplete CD169^+^ cells, CD169-DTR mice (CD169^+/DTR^) were injected i.p. twice with 100 ng DTx (Sigma Chemical Co.), 1 and 2 days before infection. Statistical comparisons were by Student's two-tailed unpaired *t*-test.

### Cells and viruses

BHK-21 cells (ATCC CCL-10), NIH-3T3 cells expressing Cre (3T3-Cre) (Stevenson *et al.*, 2002) and NIH-3T3 cells expressing the MuHV-4 ORF50 transcriptional transactivator under a doxycycline-dependent promoter (3T3-50) (Milho *et al.*, 2009) were grown in Dulbecco's modified Eagle's medium with 2 mM glutamine, 100 IU penicillin ml^− 1^, 100 μg streptomycin ml^− 1^ and 10 % FCS (PAA Laboratories). All viruses were derived from a BAC-cloned MuHV-4 genome (Adler *et al.*, 2000), removing the loxP-flanked BAC cassette by passage in 3T3-Cre cells. Fluorochrome-switching (MHV-RG) (Frederico *et al.*, 2012), luciferase-expressing (MHV-LUC) (Milho *et al.*, 2009), ORF50-deficient (Milho *et al.*, 2009) and EF1α promoter-driven eGFP (MHV-GFP) MuHV-4 derivatives (May & Stevenson, 2010) have been described previously. ORF50^− ^ MuHV-4 was grown and titrated in 3T3-50 cells treated with doxycycline (1 μg ml^− 1^). To make virus stocks, cell-free virions were recovered from infected-cell supernatants by ultracentrifugation (13 000 *g*, 2 h). Any cell debris was removed by low-speed centrifugation (500 *g*, 5 min) and filtration (0.45 μm). All viruses were stored at − 80 °C.

### Infectivity assays

Infectious virus was measured by plaque assay (de Lima *et al.*, 2004): dilutions of virus stocks or organ homogenates were incubated with BHK-21 cells (2 h, 37 °C), overlaid with 0.3 % carboxymethylcellulose, cultured for 4 days and then fixed (4 % formaldehyde) and stained (0.1 % toluidine blue) for plaque counting. Total recoverable (lytic plus reactivable latent) virus was measured by infectious centre assay (de Lima *et al.*, 2004): single-cell suspensions of freshly isolated LNs or spleens were plated onto BHK-21 cell monolayers and then overlaid, cultured, fixed, stained and plaque counted as above. To assay fluorochrome switching by MHV-RG, plaque or infectious centre assays were performed at limiting dilutions in 96-well plates (12–24 wells per dilution), and plaques were scored as red or green fluorescence under UV illumination after 4 days.

### Immunofluorescence

Organs were fixed in 1 % formaldehyde, 10 mM sodium periodate, 75 mM l-lysine (24 h, 4 °C), equilibrated in 30 % sucrose (18 h, 4 °C) and then frozen in OCT. Sections (6 μm) were air dried (1 h, 23 °C), blocked with 0.3 % Triton X-100, 5 % normal goat serum (1 h, 23 °C) and then incubated (18 h, 4 °C) with combinations of primary antibodies to eGFP [rabbit polyclonal Ab (pAb); Abcam], MuHV-4 (rabbit pAb, raised in house against whole virus), CD169 (rat mAb 3D6.112; Serotec), CD45RB/B220 (rat mAb RA3-6B2; Abcam), CD68 (rat mAb FA-11; Biolegend), ER-TR7 (rat mAb; AbCam) and CD11c (hamster mAb N418). ZsGreen1 fluorescence was visualized directly. For CD11c staining, fixed sections were first treated with proteinase K (10 μg ml^− 1^; 5 min, 23 °C). Sections were washed three times in PBS, incubated (1 h, 23 °C) with combinations of Alexa Fluor 488-conjugated goat anti-rabbit IgG pAb, Alexa Fluor 94-conjugated goat anti-hamster IgG pAb and Alexa Fluor 568-conjugated goat anti-rat IgG pAb (Invitrogen), washed three times in PBS and mounted in Prolong Gold+DAPI. Fluorescence was visualized with a Leica TCS SP5 or Zeiss LSM 510 confocal microscope, or a Nikon epifluorescence microscope, and analysed with ImageJ. For immunohistochemical staining, endogenous peroxidase activity was quenched in 3 % H_2_O_2_ in PBS (10 min, 23 °C). Sections were blocked with an avidin–biotin blocking kit (Vector Laboratories) and 2 % BSA, 2 % rabbit serum in PBS (1 h, 23 °C). Viral antigens were then detected with anti-MuHV-4 pAb (1 h, 23 °C), washed three times in PBS, incubated (1 h, 23 °C) with biotinylated goat anti-rabbit IgG pAb (Vector) and Vectastain Elite ABC Peroxidase complexes, washed three times in PBS, and developed with ImmPact diaminobenzidine substrate (5 min, 23 °C; Vector). Sections were counterstained with Mayer's haemalum (Sigma Chemical Co.), dehydrated in ethanol and mounted in DPX (BDH).

### Viral genome quantification

DNA was extracted from organs (Nucleospin Tissue kit; Macherey-Nagel). MuHV-4 genomic coordinates 4166–4252 (M2 gene) were amplified from the extracted DNA (50–80 ng) by quantitative PCR (Rotor Gene 3000; Corbett Research). The PCR products were quantified by hybridization with a Taqman probe (genomic coordinates 4218–4189) and converted to genome copies by comparison with a standard curve of cloned plasmid template amplified in parallel (Gaspar *et al.*, 2011). Cellular DNA of the same samples was quantified in parallel by amplifying part of the adenosine phosphoribosyltransferase (APRT) gene.
